# Spontaneous atraumatic fracture of a cervical vertebra in tuberculosis: a case report

**DOI:** 10.1186/1752-1947-6-138

**Published:** 2012-05-31

**Authors:** Sumit Gupta, Satya Ranjan Patra, Asmita Parihar

**Affiliations:** 1 Lady Hardinge Medical College, Pocket A 45 B Dilshad Garden, New Delhi, 110095, India; 2 Dr Ram Manohar Lohia Hospital, New Delhi, 110001, India; 3 University College of Medical Sciences and G.T.B. Hospital, New Delhi, 110092, India

## Abstract

**Introduction:**

Spontaneous pathological fractures of the cervical spine due to tuberculosis are rare. But with escalating incidences of atypical presentations of tubercular disease, clinicians should exercise a high index of suspicion for early diagnosis of such cases.

**Case presentation:**

We present a case of a 50-year-old Hindu man from northern India, who complained of pain and stiffness in his neck. His radiographs showed a fracture in his second cervical vertebral body. But further investigations raised the suspicion of an infective pathology, which was corroborated by magnetic resonance imaging and fine needle aspiration cytology. His symptoms improved and the fracture healed following antitubercular chemotherapy and immobilization.

**Conclusion:**

In endemic regions like India, clinicians should be on the lookout for atypical presentations of tuberculosis. Any suspicious lesion should be evaluated with care for clinical, radiological and laboratory evidences of the infection. The affected spine should be protected and appropriate chemotherapy should be instituted at the earliest opportunity.

## Introduction

Spinal involvement by tuberculosis, though very rare in developed nations, is a frequent phenomenon in developing countries like India. Prompt diagnosis of the disease is of critical importance not only for the prevention of neurological complications but also for the immense economic burden it may create on the patient and his or her family. Atypical clinical presentations of spinal tuberculosis are growing in incidence in recent decades. They may be associated with unusual locations in the spine, atypical clinical signs and symptoms as well as atypical radiological pictures. The epidemiological association of tuberculosis with human immunodeficiency virus has increased the fatality rate of tuberculosis by many folds, making it one of the most dreaded health hazards in the developing nations.

A high index of suspicion is often required for early recognition of an atypical case. We present an unusual case of spinal tuberculosis involving the second cervical vertebra, which presented as a spontaneous fracture of the vertebral body and posed difficulty in diagnosis.

## Case presentation

A 50-year-old Hindu man from northern India presented with pain in his neck and restriction of neck movements of two months duration. The pain was not radiating to any other part, was present throughout the day and was aggravated during the night and after activity. He had no history of any traumatic episode. Our patient was experiencing little relief with analgesics. The pain was not associated with fever; he had no weakness in any of his limbs, nor difficulty in speech or deglutition. On examination, he had tenderness over the spinous processes of the upper half of his cervical spine along with spasm of his neck muscles. There was no deformity or gibbus. Our patient had gross restriction of motion of his cervical spine in all directions. He had no palpable lymph nodes in his neck. On neurological examination, there was no deficit in any limb and his tendon reflexes were normal. He had no other systemic illness.

Laboratory investigations showed little abnormality other than an increased erythrocyte sedimentation rate (54 mm in the first hour).

**Figure 1:**
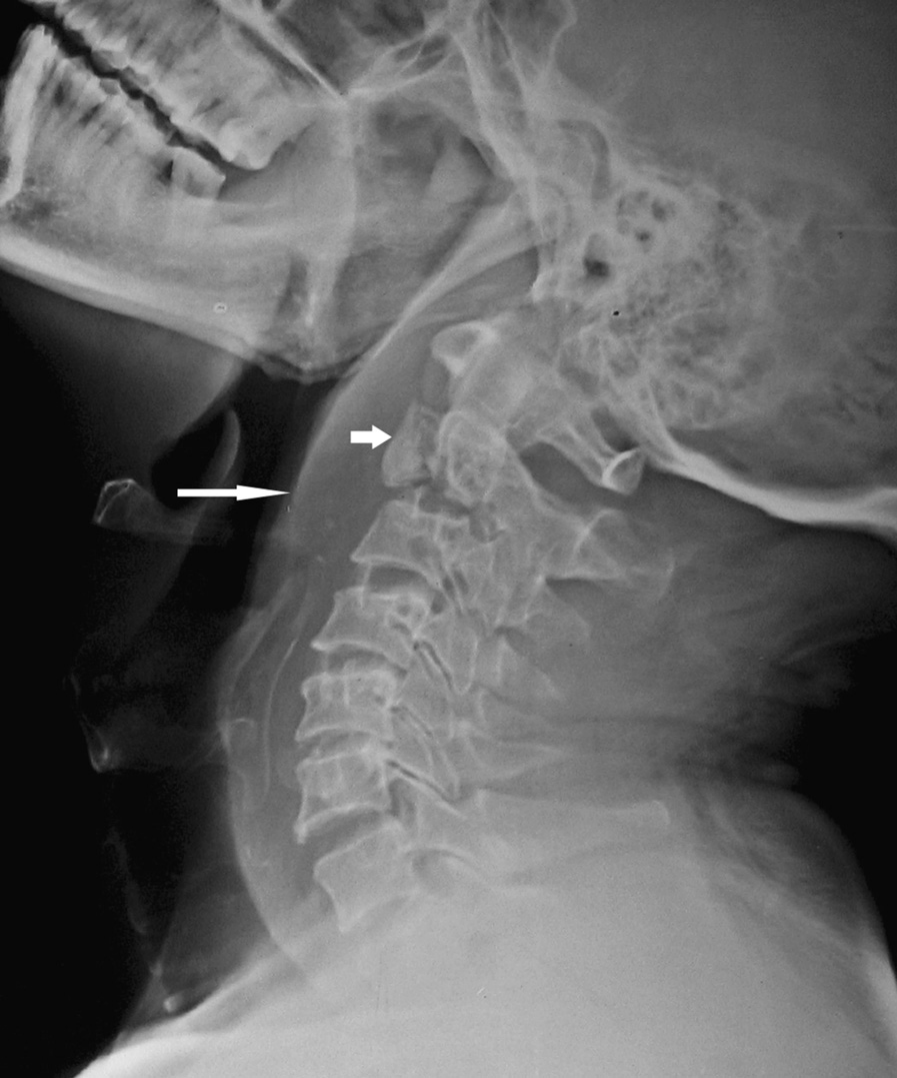
Lateral radiograph of the cervical spine shows a fracture in the body of the C2 vertebra (small arrow) with increased soft tissue shadow in the prevertebral region (long arrow).

A lateral view of plain radiographs showed a fracture of the body of his second cervical (C2) vertebra with mild displacement of the fractured anterior body fragment. The odontoid process appeared to be in normal alignment with his C1 vertebra. There was also a significantly increased prevertebral soft tissue shadow anterior to the C1, C2 and C3 vertebral region (Figure 1), which indicates the presence of a retropharyngeal abscess.

**Figure 2:**
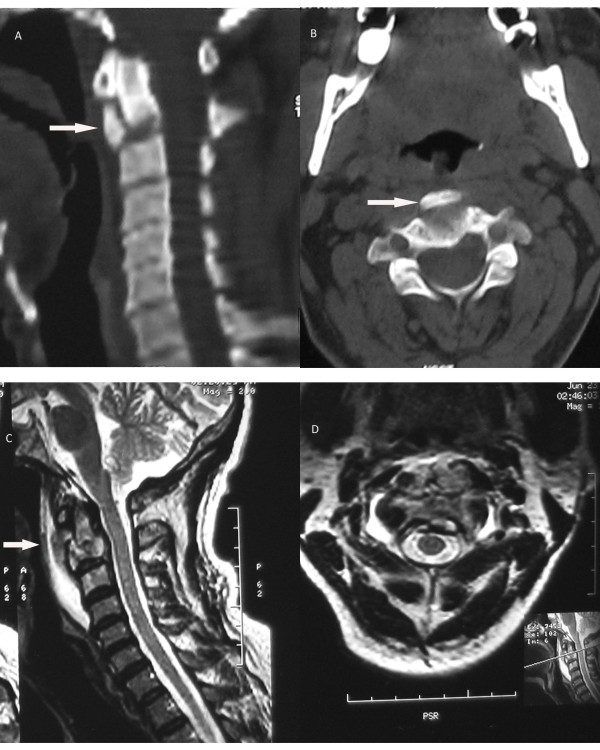
**Computed tomography images.(A)** Saggital cut of the computed tomography scan depicting the anterior fractured fragment of the C2 vertebral body (shown by arrow). **(B)** Transverse cut of the same region and the fractured anterior fragment (arrow). **(C)** Saggital magnetic resonance image (T2-weighted) showing destruction of the C2 vertebral body as well as the large prevertebral collection (arrow). **(D)** Transverse image of the same region.

A computed tomography scan showed fragmentation of his C2 vertebral body and the anterior fragment lying separate from the parent bone. Posterior elements were found to be normal (Figure 2A, B).

MRI clearly depicted the extent of vertebral involvement in T1-weighted, T2-weighted and fat suppression sequences. The destruction and expansion of the C2 vertebral body was seen along with significant pre- and paravertebral collection. This appeared hypointense in T1-weighted and hyperintense on T2-weighted images. The soft tissue mass was seen compressing the airway anteriorly and was causing slight indentation of the thecal sac posteriorly. However, the cord appeared normal on signal intensity. His C2 and C3 vertebral bodies appeared hyperintense on fat suppression images, suggesting extensive bone marrow edema. His cervical intervertebral discs appeared degenerated at various levels but otherwise appeared intact (Figure 2C, D).

**Figure 3:**
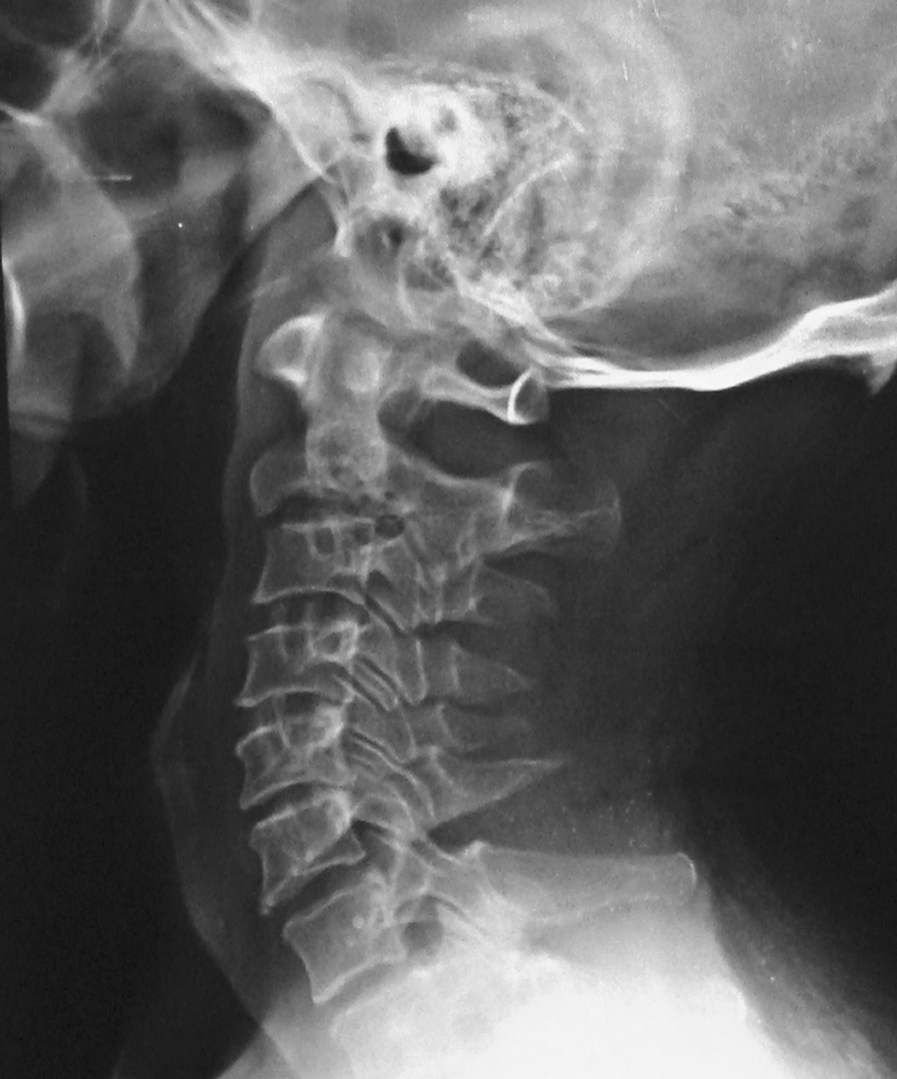
The follow-up lateral radiograph shows good healing of the C2 vertebral body; the prevertebral soft tissue shadow has also returned to normal.

Transoral fine needle aspiration cytology of the lesion yielded caseous material on cytology but did not show any acid-fast bacilli. Our patient was given antitubercular treatment with a four-drug regimen (rifampicin, isoniazid, ethambutol, pyrazinamide) for two months followed by a two-drug regimen (rifampicin, isoniazid) for a period of four months. His cervical spine was protected by a Philadelphia collar. On follow-up radiographs, the fracture in his C2 vertebra was found to be united by the end of 16 weeks and the prevertebral soft tissue shadow returned to its normal limits (Figure 3). The pain and stiffness in his neck also improved significantly following treatment, although some terminal restriction of motion remained even at the end of 25 months follow-up.

## Discussion

Tuberculosis has been implicated for causing spontaneous fractures of bones like the sternum, femur, humerus, ulna, ribs, clavicle and dorsolumbar spine [1-6]. But tubercular spontaneous fracture of upper cervical vertebra is very unusual.

Silent fractures of the sternum or ribs due to tuberculosis have been described to occur after paroxysmal bouts of cough, often due to concomitant pulmonary disease [7]. Tuberculosis affecting the upper cervical spine usually results in the destruction of the atlantoaxial articulation, varying the degree of subluxation and, ultimately, may lead to cord compression and even death [8]. Presenting symptoms of cervical spine tuberculosis may range from just pain and stiffness to torticollis, spinal deformity, headache, hoarseness of voice, dysphagia or neurological deficit of varying severity [8]. Radiologic findings may include mild prevertebral increased soft tissue shadow to wedge compression of the vertebral body, atlantoaxial dislocation or widespread destruction of vertebral elements.

Our patient had minimal symptoms at the time of his presentation without any neurologic involvement or constitutional symptoms. He was not involved in any trauma, but radiographs showed a fracture of his C2 vertebral body in the coronal plane. The only radiographic finding hinting towards an infective pathology was the increased prevertebral soft tissue shadow. Computed tomography and MRI findings were able to depict the widespread involvement of the bone and surrounding soft tissues by the disease process. But the disc spaces adjacent to the affected vertebrae were uncharacteristically preserved.

However, silent spontaneous fractures of cervical vertebrae have also been attributed to conditions other than tuberculosis, such as fibrous dysplasia or primary and secondary tumors [7,8]. Therefore, such clinical and radiological findings should also be carefully differentiated from pathologies like malignancy or fibrous dysplasia.

Fibrous dysplasia involving the cervical spine is extremely rare in occurrence. It is usually seen in the polyostotic variety of the disease with more generalized involvement of the skeletal system. Fractures in other bones and *café au lait* spots may be the other associated features. Patients with spinal fibrous dysplasia may present with an aching pain or deformity. Only a little restriction in motion is seen in most cases, but this may be relative to the severity of the spinal involvement and the degree of neurological deficit. On radiographs, fibrous dysplasia normally presents as expansile, lytic lesions with ground-glass appearance and thinning of cortices (although other radiographic patterns have also been described). The MRI picture may vary depending on the fibrous, cartilaginous or hemorrhagic content of the lesions. Soft tissue involvement and pre- or paravertebral collections are not associated with this pathology.

Metastatic tumors of the cervical spine may mimic tubercular pathology but are rare in incidence. The most common primary sites are the breast or prostate [9,10]. Their presence may be silent until the tumor spreads beyond the vertebral body and neurologic compression, pathological fracture or spinal instability occurs. Such a lesion in the second cervical vertebra may prove fatal with a pathological fracture. A high index of clinical suspicion is of critical importance in patients with a history of breast carcinoma because the radiological evidence is often seen to lag behind the clinical stage of the disease [9]. Symptomatic patients may present with unremitting neck pain and restriction of neck motion (particularly rotation). Bone scans may be of great value in such cases to pick up early metastatic lesions. Evidence of the destruction of the pedicles on radiographs is the characteristic sign of metastatic disease. Computed tomography and MRI may also be useful in differentiating metastatic tumors from infective pathologies.

## Conclusion

Study of this case emphasizes the need for careful assessment by the clinician of various unusual atypical presentations and symptomatology of spinal tubercular disease. The disease process and progression may not necessarily follow the classical textbook pattern of tubercular infection. The imaging modalities also may not reveal the characteristic bone and soft tissue changes associated with tuberculosis of spine. As in our case, the commonly encountered paradiscal pattern of involvement of two adjacent vertebrae is not always witnessed. The affected spine should be protected and the fracture may unite with conservative treatment only.

## Consent

Written informed consent was obtained from the patient for publication of this case report and accompanying images. A copy of the written consent is available for review by the Editor-in-Chief of this journal.

## Competing interests

The authors declare that they have no competing interests.

## Authors’ contributions

SG and SP analyzed and interpreted the patient data. AP performed the fine needle aspiration cytology of the prevertebral swelling, and was a major contributor in writing the manuscript. All authors read and approved the final manuscript.
